# Quantitative image analysis of microbial communities with BiofilmQ

**DOI:** 10.1038/s41564-020-00817-4

**Published:** 2021-01-04

**Authors:** Raimo Hartmann, Hannah Jeckel, Eric Jelli, Praveen K. Singh, Sanika Vaidya, Miriam Bayer, Daniel K. H. Rode, Lucia Vidakovic, Francisco Díaz-Pascual, Jiunn C. N. Fong, Anna Dragoš, Olga Lamprecht, Janne G. Thöming, Niklas Netter, Susanne Häussler, Carey D. Nadell, Victor Sourjik, Ákos T. Kovács, Fitnat H. Yildiz, Knut Drescher

**Affiliations:** 1 Max Planck Institute for Terrestrial Microbiology, Marburg, Germany; 2 Department of Physics, Philipps-Universität Marburg, Marburg, Germany; 3 Department of Microbiology and Environmental Toxicology, University of California, Santa Cruz, CA USA; 4 Bacterial Interactions and Evolution Group, Department of Biotechnology and Biomedicine, Technical University of Denmark, Kongens Lyngby, Denmark; 5 Institute for Molecular Bacteriology, TWINCORE, Centre for Experimental and Clinical Infection Research, Hannover, Germany; 6 Department of Clinical Microbiology, Copenhagen University Hospital, Rigshospitalet, Copenhagen, Denmark; 7 Department of Biological Sciences, Dartmouth College, Hanover, NH USA; 8 Zentrum für Synthetische Mikrobiologie, SYNMIKRO, Marburg, Germany; 9 Present Address: Eawag, Swiss Federal Institute of Aquatic Science and Technology, Dubendorf, Switzerland

**Keywords:** Biofilms, Microbial communities, Computational platforms and environments, Image processing

## Abstract

Biofilms are microbial communities that represent a highly abundant form of microbial life on Earth. Inside biofilms, phenotypic and genotypic variations occur in three-dimensional space and time; microscopy and quantitative image analysis are therefore crucial for elucidating their functions. Here, we present BiofilmQ—a comprehensive image cytometry software tool for the automated and high-throughput quantification, analysis and visualization of numerous biofilm-internal and whole-biofilm properties in three-dimensional space and time.

## Abstract (web summary)

BiofilmQ is an image cytometry software tool that enables the visualization, quantification and analysis of biofilm properties, providing insights into their structure and function.

## Main

Spatially structured microbial communities display spatial gradients of nutrients and other diffusible molecular compounds, as well as spatiotemporal variation in species composition and cellular differentiation^1–4^. For biofilm phenotyping, as well as for characterizing phenotypes of particular cells within biofilms, it is critical to be able to perform image-based quantitative measurements of fluorescent reporters and structural features for particular regions inside three-dimensional (3D) biofilms.

Extracting the desirable information from 3D images relies on non-trivial automated image analysis. The most widely used tool for biofilm image analysis in the literature is COMSTAT^5,6^, which provided one of the first tools to objectively determine differences in biofilm morphology. COMSTAT and the alternative software tools for biofilm image analysis^7–9^ have been tremendously important for biofilm research by providing parameters for 3D phenotyping, yet they are not designed for analysing biofilm internal properties with spatial resolution, and they do not include the functionality to visualize data. Image analysis tools developed for microbial ecology^10–12^ have the ability to measure alternative parameters, including, morphology analysis^10^ and 3D correlation functions^12^. The design of biofilm research projects and the discovery of new biofilm behaviours are presently limited by the lack of modern cytometry software tools that can quantify a comprehensive set of spatially and temporally resolved structural parameters and fluorescent reporters inside 3D biofilms, and visualize the resulting data.

Powerful and user-friendly tools that have recently enabled quantitative analyses for bacterial cell biology for two-dimensional (2D) images^13–16^. Inspired by these tools, we have integrated algorithms for image analysis of the internal properties of 3D microbial communities with data analysis and data visualization capabilities in the form of a software tool, BiofilmQ (https://drescherlab.org/data/biofilmQ), which provides a graphical user interface and requires no knowledge of programming. BiofilmQ is built on the basis of standard image analysis techniques, as well as new algorithms for image cytometry and object tracking, of which technical descriptions are provided in the online documentation. Extensive documentation and video tutorials guide users through each step in the image analysis, data analysis and data visualization workflow. Here we describe the concept, capabilities and limitations of BiofilmQ and demonstrate its usefulness for the quantitative characterization of microbial communities.

**Fig. 1:**
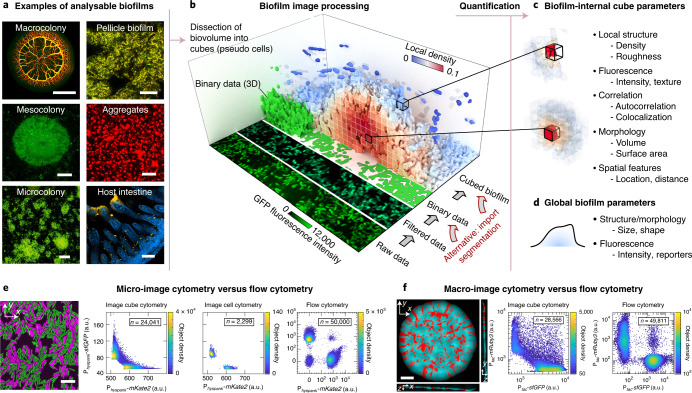
Quantification of microbial community properties with spatial resolution in BiofilmQ. **a**, Examples of different biofilm image categories that can be analysed using BiofilmQ: *E. coli* macrocolony, *V. cholerae* meso- and microcolonies, *Bacillus subtilis* pellicle and floating aggregates, *V. cholerae* biofilm (yellow) on mouse intestinal villi (blue). Many file formats are supported, based on the Bio-Formats toolbox^36^. Scale bars, 1 mm (top left), 30 µm (middle and bottom left, and top right), 40 µm (middle right), 100 µm (bottom right). **b**, The BiofilmQ image processing pipeline for a 3D biofilm image. The raw fluorescence image is filtered and thresholded to obtain a binary representation of the biofilm. These 3D binary data are then dissected into cubes of a user-defined size, and the cubes are then used to quantify the biofilm properties. Alternatively, binary images of the biofilm or single cells can be imported. Here, each cube in the biofilm is coloured according to the local biovolume density, which is one of the cube properties that can be extracted. **c**,**d**, Many parameters can be quantified for each cube (**c**) and for the whole biofilm (**d**). **e**, A *B. subtilis* microcolony (left) on an agar pad consisting of two strains (a strain constitutively expressing superfolder green fluorescent protein (sfGFP) and a strain constitutively expressing mKate2) was analysed using cube-based image cytometry, single-cell image cytometry and flow cytometry. The image cytometry results are qualitatively similar, and the flow cytometry results also show two additional cell populations (non-fluorescent cells and cells with fluorescence in both channels). The data shown are one example out of *n* = 3 experiments, which all showed the same qualitative results. Scale bar, 10 µm. **f**, For an *E. coli* macrocolony (left) on agar consisting of two strains (a strain constitutively expressing sfGFP and a strain constitutively expressing mRuby3), we compared the results from cube-based image cytometry and flow cytometry. The data shown are one example out of *n* = 3 experiments, which all showed the same qualitative results. Scale bar, 500 µm. a.u., arbitrary units.

**Fig. 2:**
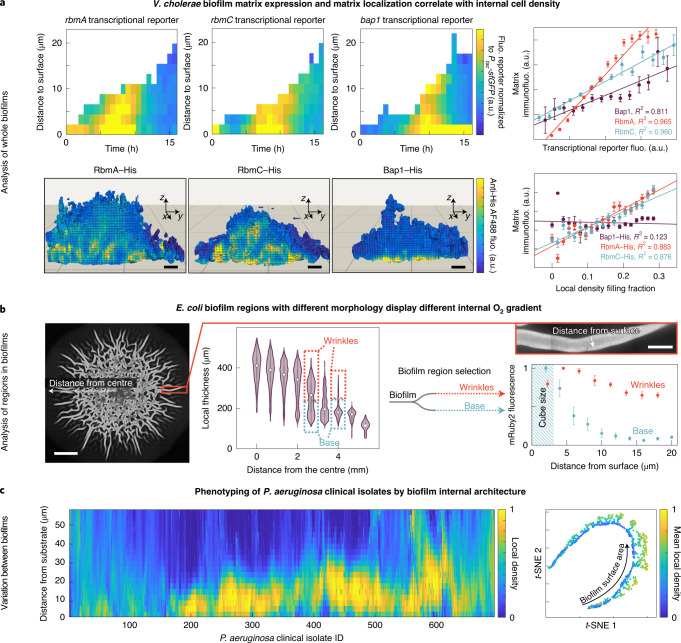
Applications of spatial image cytometry for characterizing biofilm biology. **a**, By analysing biofilm spatiotemporal development of whole *V. cholerae* microcolonies in flow chambers, the transcription of key matrix biosynthesis genes was correlated with matrix localization and the cell density structure inside biofilms. Top left, the space–time kymographs of reporters for *rbmA*, *rbmC* and *bap1* transcription were normalized to the signal of constitutively expressed *sfGFP*. Representative of *n* = 3 independent biofilms for each reporter. Top right, for biofilms grown to a particular timepoint (15 h), the correlation of the spatial distribution of transcriptional reporters and matrix localization immunofluorescence signal was analysed. Data are mean ± s.e.m. across 100–300 cubes for one biofilm. Representative of *n* = 3 independent biofilms for each reporter. Bottom left, renderings of the immunofluorescence localization for biofilms grown up to 15 h show a characteristic biofilm internal spatial distribution for each matrix component. Scale bars, 2 µm. Bottom right, RbmA and RbmC show a high correlation (*R*^2^) with the biofilm internal cell density, measured as the local density filling fraction per cube. Data are mean ± s.e.m. across 100–300 cubes for one biofilm, representative of *n* = 3 independent biofilms for each reporter. Fluo., fluorescence. **b**, For *E. coli* macrocolonies on agar, cube cytometry was used to measure the distribution of the local biofilm thickness, revealing quantitative signatures of the wrinkles and the flat biofilm base. By analysing the wrinkles and the base as different subpopulations (shown for one colony, representative for *n* = 3 colonies), the spatial distribution of fluorescence patterns was measured in the wrinkles and base. Data are mean ± s.e.m. between *n* = 5 different wrinkles and *n* = 6 different 400 µm^2^ regions of the biofilm base. Here, the mRuby2 fluorescence was used as a proxy for O_2_ penetration, as mRuby2 requires O_2_ to fold into a fluorescent conformation. Scale bars, 2 mm (left) and 100 µm (inset). **c**, For 694 *P. aeruginosa* wild-type clinical isolates, the 3D internal biofilm architecture was analysed (*n* = 2 biofilm images for each strain), resulting in 420 parameters for each strain. The spatial distribution of one structural parameter (the local density) is shown here for each strain in the heat map. Dimensionality reduction using *t*-distributed stochastic neighbour embedding (*t*-SNE) revealed that the strains primarily differ in two biofilm structural parameters (mean local density and biofilm surface area). Source data

BiofilmQ is designed for analysing fluorescence images of a wide variety of spatially structured microbial communities and growth geometries, including microscopic, mesoscopic, macroscopic colonies and biofilms on surfaces, and free-floating aggregates as well as communities in the context of eukaryotic hosts. Microbial communities can be analysed irrespective of the size, growth geometry, morphology, species or the number of fluorescence channels (Fig. 1a). The only requirement for BiofilmQ is that the software must be able to identify the biovolume of the biofilm using one fluorescence channel or using an imported segmentation. Biofilm biovolume detection is an example of semantic segmentation in image analysis^17^, and different segmentation algorithms have received considerable attention in the biofilm literature^18–24^, as the segmentation quality can have a large impact on the analysis results. To perform accurate biofilm segmentation for a wide variety of image types and signal levels (Fig. 1a), BiofilmQ includes the following three different segmentation options: (1) automatic segmentation using classical algorithms, such as Otsu^25^, Ridler–Calvard^26^, robust background or maximum correlation thresholding^27^; (2) semi-manual thresholding supported by immediate visual feedback; and (3) import of presegmented images into BiofilmQ. If users choose to import presegmented images, we recommend general-purpose segmentation tools (such as ilasik^28^) or convolutional neural networks (such as U-Net^29^), which can be trained for particular image types, fluorescence levels or biofilm morphologies to give very high segmentation accuracy. After the segmentation of the biofilm biovolume, we recommend visual inspection of the segmentation accuracy, which is displayed by BiofilmQ. The automated and semi-manual segmentation options that are provided yielded a good segmentation accuracy for the different types of biofilm images that are shown in Figs. 1 and 2. Thus, the main focus of BiofilmQ is the cytometry, data analysis and data visualization after the segmentation step.

To quantify properties inside microbial communities with spatial resolution, BiofilmQ can use images with single-cell resolution or lower resolution. For images in which single-cell segmentation is not possible, BiofilmQ can dissect the biofilm biovolume into a cubical grid, with a user-defined cube size (Fig. 1b). For low-resolution images, the cubes correspond to multicell regions inside the biofilm. However, for images with spatial resolution that is close to single-cell resolution, the cube size can be chosen to be approximately equal to the cell volume such that the cubes can be conceptualized as pseudocell objects, even though the cubes typically do not align with the cells, and a cube might not contain only a single cell. For each cube, numerous cytometric properties and the spatial context are computed (Fig. 1c,d), enabling 3D spatially resolved quantification of the internal properties of the biofilm for images that range from microcolonies up to millimetric macrocolonies. For microscopic colonies in which single cells can be distinguished, cube-based image cytometry gives results that are similar to single-cell image cytometry and flow cytometry (Fig. 1e). For macroscopic bacterial colonies, where the imaging resolution does not permit single-cell image cytometry, the cube-based image cytometry also results in data that are similar to data from single-cell flow cytometry analyses (Fig. 1f). However, in contrast to flow cytometry, image cytometry offers the possibility to quantify properties in the spatial and temporal context directly inside living biofilms, which is utilized by BiofilmQ extensively. Generally, a cube size (and image resolution) must be chosen that is appropriate for the biological process under investigation—which is not necessarily the length scale of single cells.

**Extended Data Fig. 1:**
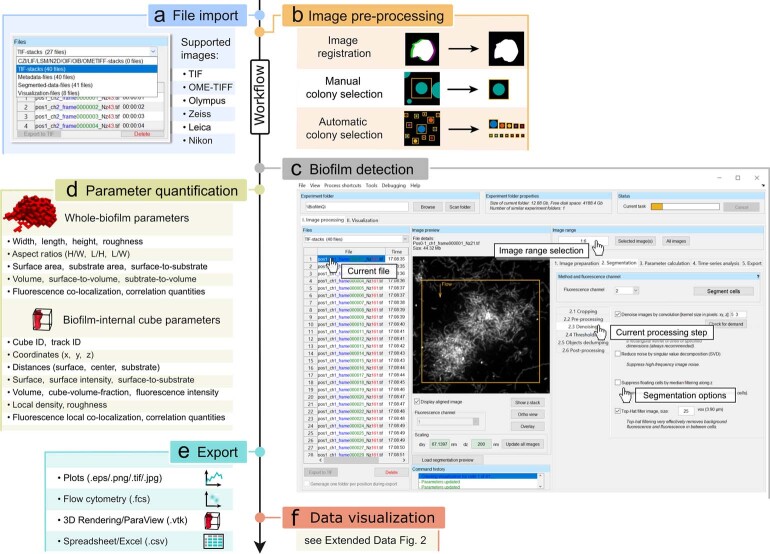
Workflow of the BiofilmQ graphical user interface for image processing and parameter quantification. **a**, First, images have to be imported; a wide range of formats are supported, including 3D image formats, and TIF image sequences, based on the Bio-Formats toolbox^36^. **b**, Next, optional pre-processing steps including image time series registration, filtering for noise reduction, and colony separation can be performed to improve the segmentation results. **c**, Biomass must be distinguished from background, which can be performed automatically using different thresholding algorithms, or semi-manually, or by importing a segmented binary image from other image analysis tools. After the segmentation of the biofilm volume, the biofilm can be dissected into cubes of a user-defined size, or a single-cell segmentation can be imported. **d**, Quantitative characterization of the biofilm is achieved *via* the parameter calculation for the biofilm as a whole, and for each cube in the biofilm. **e**, Parameter quantifications and biofilm structural analysis can be exported either as spreadsheets, flow-cytometry data format, graphs, or as input data for a 3D rendering software. **f**, The extensive data visualization capabilities that are built into BiofilmQ are described in Extended Data Fig. 2. Key steps in the BiofilmQ workflow are further described in Supplementary Note 1.

A limitation of the cube-based cytometry is that, for bacterial communities with different cell sizes, the average number of cells per cube may vary within the community. To overcome this limitation, users can import custom-segmented biofilm images (Fig. 1b), for example, with their own single-cell segmentation^21,22,24,30^. Convolutional neural networks are rapidly improving in segmentation accuracy at present, and it is probable that they will result in highly accurate 3D single-cell segmentation for images with sufficiently high resolution in the near future. The cytometry and data analysis workflow of BiofilmQ (Extended Data Fig. 1, Supplementary Note 1) can be performed using any segmented object that is imported, or using the cubical objects that are built in by default. However, for simplicity, we hereafter refer to the objects on which cytometric quantifications are based as ‘cubes’. Although BiofilmQ was originally developed for 3D image analysis, it can also analyse 2D images.

**Extended Data Fig. 2:**
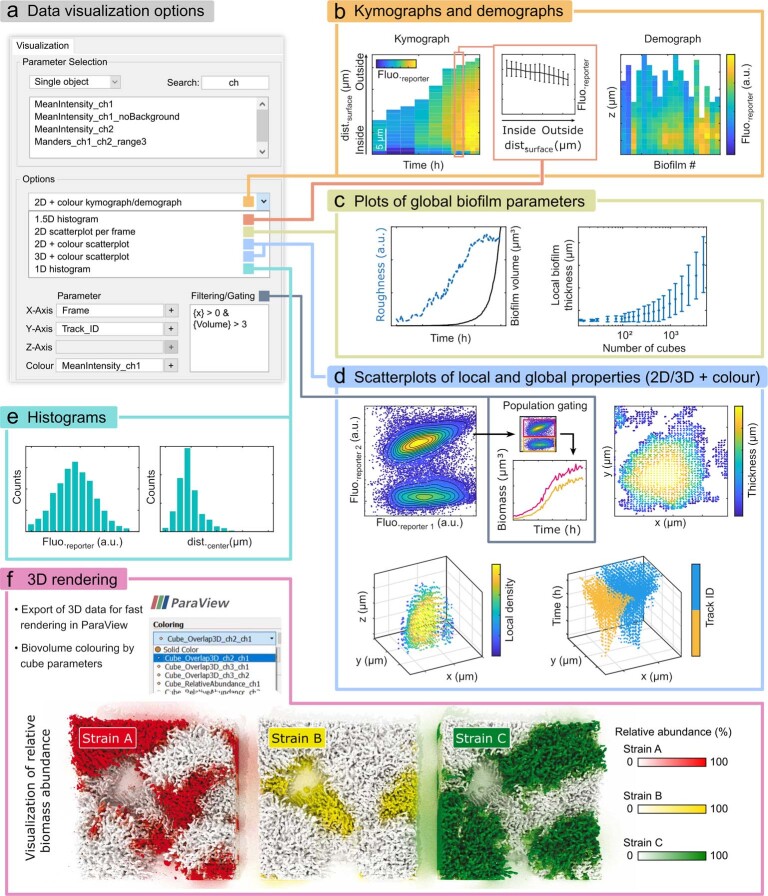
Examples of the data visualization and plot categories that are possible within BiofilmQ. All graphs shown in this figure were produced directly by BiofilmQ, except panel **f**. **a**, Screenshot showing several key elements of the data visualization tab in the BiofilmQ graphical user interface, depicting how to choose the axis of figures to be plotted, and the plot type. **b**, Left, a kymograph quantifies fluorescent reporter expression as a space-time heatmap for *n* = 1 biofilm. In this example, the fluorescence of an RpoS-mRuby3 translational fusion is plotted over time and space during *V. cholerae* C6706 WT biofilm development. Centre, a 1.5D histogram shows the relation between fluorescent reporter intensity and position in the biofilm for a single time-point, mean values are plotted with error bars representing the standard deviation of 100–1500 cubes with similar distance from the surface. Right, the heatmap represents a demograph of *n* = 19 different biofilms, which reveals spatially-resolved differences between biofilms, for a particular cube-level parameter (here: RpoS-mRuby3 fluorescence as a function of height in *V. cholerae* biofilms after 18 h of growth). **c**, Left, several global biofilm parameters can be plotted into the same 2D graph for better comparison. In this example, the biofilm roughness and volume during biofilm development of a *V. cholerae* N16961 rugose Δ*crvA* strain are plotted for *n* = 1 biofilm. Right, to analyze the behavior of a parameter in a time series, other time-related quantities, such as the number of cubes in the biofilm, can be used as the timescale on the x-axis for the same biofilm as in the left panel; showing mean +/- error bars represent std. dev. of all of the 3000–5000 cubes at a given time point. **d**, Top left, analogous to flow cytometry, BiofilmQ can perform biofilm image cytometry, comparing two fluorescent reporters or any other cube parameter. In this example, results are shown from a biofilm co-culture of two *V. cholerae* N16961 WT strains that constitutively produce sfGFP (*n* = 1 dataset of two biofilms merging during growth); one of these strains additionally produces mRuby2 constitutively. The segmentation was performed on the sfGFP channel. The gating/filtering option enables the separation of two populations; properties of each gated population can then be visualized separately. Top right, in a 2D+colour scatter plot, extracted cube parameters can be visualized; this example shows the biofilm thickness distribution in space at a specific timepoint (22 h) during the development of *n* = 1 biofilm of *V. cholerae* C6706 WT. Bottom left, a 3D+colour scatterplot visualizes quantified cube parameters, but provides one additional axis. In this example, the spatial distribution of the local density during *V. cholerae* C6706 WT biofilm growth is shown at a particular timepoint (12 h, *n* = 1 biofilm). Bottom right shows another example of a 3D+colour plot, visualizing two tracked *V. cholerae* N16961 rugose Δ*crvA* biofilm colonies with the same constitutive fluorescent protein expression (sfGFP), growing together over time (*n* = 1 dataset of two biofilms merging during growth). Separation of lineages is performed *via* cube tracking. **e**, Histograms of quantified cube parameters: These examples show the fluorescence signal of an RpoS-mRuby3 translational fusion reporter (left) and the distance of each cube to the biofilm center (right), for a *V. cholerae* C6706 WT *n* = 1 biofilm grown for 22 h. **f**, The location and relative abundance of three different *V. cholerae* N16961 rugose strains (differing by the colour of a constitutively expressed fluorescent protein marker: mTFP1, mKOκ, mKate2) were quantified using BiofilmQ and the result was exported and rendered in 3D with the ParaView software for visualization.

The internal parameters of the biofilm are quantified for each cube and therefore have a 3D spatial and potentially a temporal dependence (Extended Data Fig. 1d). The location of each cube can be expressed as the distance to the biofilm outer surface, to the substratum, to the centre of mass of the biofilm volume or the centre of mass of the biofilm volume projected onto the substratum. For each cube, a total of 49 structural, textural and fluorescence properties, as well as correlations between fluorescence channels and density can be calculated (Supplementary Table 1). Furthermore, users may define custom combinations of parameters as new parameters directly inside the graphical user interface. It is also possible to track cube lineages (Supplementary Note 2) to measure clonal cluster sizes and similar properties. Analogous to flow cytometry, the biofilm image cytometry provided by BiofilmQ enables users to apply gates/filters to their data for each cube to effectively select cube subpopulations (Fig. 2b, Extended Data Fig. 2d, inset).

In addition to the spatially resolved internal parameters of the biofilm described above, BiofilmQ also calculates hundreds of parameters for the whole biofilm, which we refer to as global parameters (Supplementary Tables 2 and 3 and Extended Data Fig. 1d). Some of these parameters characterize the size and morphology of the whole biofilm, including its volume, mean thickness, surface area and roughness coefficient, as well as several combinations of these values, such as the surface-to-volume ratio. A small subset of the parameters can also be quantified using COMSTAT^5,6^, for which we chose identical implementations to enable compatibility (Supplementary Table 4 and Supplementary Notes). In addition to these structural parameters, BiofilmQ can quantify correlations between different fluorescence reporters through the Manders’ overlap coefficient, Pearson’s correlation coefficient, volume overlap fractions and relative abundances of biovolume. These parameters enable, for example, quantitative measurements of species cluster sizes and species separation distances in multispecies biofilms using 3D correlation functions.

After the analysis of a single 3D (or 2D) biofilm image, BiofilmQ can apply the same analysis to a whole time series (to analyse the temporal variation of a single biofilm), or to a non-time-series collection of biofilm images (to analyse the variation within a population of biofilms). All data analysis operations can be performed in high throughput using the inbuilt batch-processing capabilities of BiofilmQ, and the results can be exported to standard formats (Extended Data Fig. 1e) or directly visualized (Extended Data Fig. 2).

In addition to the quantification of biofilm-internal and whole-biofilm parameters, another main focus of BiofilmQ is the visualization of these data to generate numerous types of editable figures. Examples of the different classes of graphs that can be created using BiofilmQ are described in the Methods and shown in Extended Data Fig. 2.

The quantification, analysis and data visualization of 3D biofilm internal parameters enabled by BiofilmQ may be used to gain insights into biofilm biology, as demonstrated below using examples of spatiotemporal biofilm development, biofilm subpopulation analysis and biofilm phenotyping.

To understand the relationship between spatiotemporal biofilm matrix gene expression, matrix localization and the resulting biofilm architecture, we imaged the development of 3D *Vibrio cholerae* microcolonies. From these images, we quantified spatiotemporal transcriptional reporters for the key matrix genes *rbmA*, *rbmC* and *bap1*, and we used immunofluorescence to quantify the abundance and location of RbmA, RbmC and Bap1 as well as the structural biovolume density inside the biofilm (Fig. 2a). We first noticed a high correlation between transcriptional reporters and extracellular matrix immunofluorescence for all three matrix proteins. We also discovered that, inside the biofilm, the abundance of the matrix proteins RbmA and RbmC is positively correlated with the cell density, yet there is no such correlation for Bap1, indicating that the different matrix proteins have different functional roles that require further investigation.

When grown on agar, many bacterial species form millimetre-sized macrocolonies that develop a wrinkly morphology. These wrinkles have been hypothesized to generate a higher access to atmospheric oxygen for the whole colony, by increasing the surface-to-volume ratio compared with non-wrinkled colonies^31^. Using macrocolonies of *Escherichia coli*, we tested this hypothesis (Fig. 2b) by detecting the wrinkles using their signature in the local thickness distribution, followed by a separate downstream subpopulation analysis for the wrinkles and non-wrinkled base of the macrocolony. As the protein mRuby2 requires oxygen to fold into a fluorescent conformation, fluorescence profiles of constitutively expressed mRuby2 can be used as a proxy for oxygen penetration. This analysis revealed substantially different oxygen penetration profiles for the two different regions of the macrocolony; wrinkles maintained a higher level of oxygen compared with the non-wrinkled base of the colony, confirming the functional benefit of the wrinkled morphology for the biofilm population.

The ability to identify phenotypic differences between wild-type isolates of which the genomes are known can enable an understanding of links between genomic plasticity and phenotypic variations. For 694 sequenced clinical isolates of *Pseudomonas aeruginosa*^32^, we analysed the spatial distribution of cell density inside biofilms grown in vitro for 48 h to discover a wide variety of different biofilm internal architecture patterns (Fig. 2c). For each isolate, a high-dimensional phenotyping space was generated by measuring 420 global and biofilm internal parameters using BiofilmQ. A low-dimensional projection using *t*-distributed stochastic neighbour embedding indicated that the biofilm phenotypes of the clinical isolates primarily differ by their biofilm surface area and the biofilm internal local density, providing a starting point for understanding genetic factors that influence the development of the *P. aeruginosa* biofilm architecture.

In summary, BiofilmQ closes a critical gap in the toolset for the spatial and spatiotemporal analysis of 3D microbial communities—it combines the quantification of many previously inaccessible biofilm-internal and whole-biofilm properties with data analysis and data visualization functionalities in a single software tool. By enabling scientists without programming expertise to generate such complex analyses, BiofilmQ provides a solid quantitative foundation for future studies of spatially structured microbial communities.

## Methods

### Bacterial strains and biofilm growth

*V. cholerae*, *P. aeruginosa*, *B. subtilis* and *E. coli* strains were routinely grown in liquid lysogeny broth (LB-Miller) at 37 °C under shaking conditions. A list of the strains and plasmids used in this study is provided in Supplementary Table 5.

Flow chamber biofilm experiments to grow microcolonies and mesocolonies were performed in M9 minimal medium supplemented with 0.5% (w/v) glucose for *V. cholerae* (Figs. 1a,b and 2a and Extended Data Fig. 2). To grow flow-chamber biofilms, microfluidics chambers of 7 mm length and 500 µm × 100 µm cross-section were used^21,22^, and a flow rate of 0.1 µl min^−1^ was set using a syringe pump (Pico Plus, Harvard Apparatus). To inoculate flow chambers, overnight cultures were back-diluted 1:200 in LB medium for *V. cholerae*, and grown to an optical density at 600 nm (OD_600_) of 0.5. This culture was then used to inoculate the flow chambers. After inoculation, cells were left for 1 h to attach to the surface before the constant flow with fresh medium was initiated.

*V. cholerae* biofilms inside the mouse intestines (Fig. 1a) were grown and imaged using confocal microscopy using the microbial identification after passive clarity technique, as described by Gallego-Hernandez et al.^33^.

To grow mixed biofilms of *V. cholerae* strains containing different fluorescent protein markers (Extended Data Fig. 2f), cultures of all three strains (carrying constitutive fluorescent protein expression constructs for mTFP1, mKOκ and mKate2 in the N16961 *vpvC*^W240R^ strain background) were inoculated in microfluidics chambers at a ratio of 1:1:1, before the constant flow of fresh medium was started.

Macrocolony biofilms of *E. coli* AR3110 were initiated by spotting 5 µl of overnight culture onto solid LB medium (1.5% agar (w/v)). Plates were sealed with parafilm and incubated for 5 d (Fig. 1a; KDE1469) at 23 °C; 18 h (Fig. 1f; KDE1029 and KDE542) at 30 °C; and 5 d (Fig. 2b; KDE679) at 28 °C before imaging.

Pellicle biofilms of *B. subtilis* NCBI3610 carrying the P_*tapA*_-*gfp* and P_*tapA*_-*mKate* transcriptional reporters on the chromosome were grown in MSgg medium^34^ without shaking at the air–liquid interface in 24-well microtitre plates for 48 h at 30 °C (Fig. 1a).

To grow free-floating aggregates of *B. subtilis*, an overnight culture of the strain KDB026 was grown in LB supplemented with 0.5 mM isopropyl-β-d-thiogalactoside (IPTG) under shaking conditions. This culture contained single cells and free-floating aggregates (Fig. 1a).

Microcolonies of *B. subtilis* 168 strains constitutively expressing sfGFP or mKate2 (KDB017 and KDB174) were grown on LB agar supplemented with 1 mM IPTG (Fig. 1e), following overnight growth and a 1:200 back-dilution and regrowth up to OD_600_ = 0.4 in LB containing 1 mM IPTG. LB plates were inoculated with 2 µl of a 1:1 mixture of both strains, and covered with a coverslip for imaging.

The 694 different clinical isolates of *P. aeruginosa* were grown in 100 µl of LB medium under static conditions in a 96-well µClear microtitre plate (Greiner), followed by the addition of Syto9 dye (Thermo Fischer Scientific) to a final concentration of 2.1 µM. Confocal imaging of 48-hour-old biofilms was performed as described by Thöming et al.^32^.

### Imaging

For spatiotemporal measurements of different reporters and for separating different populations in flow chambers, biofilms were imaged with a Yokogawa CSU confocal spinning-disk unit mounted onto a Nikon Ti-E inverted microscope using a Plan Apo ×60/1.4 NA oil-immersion objective (Nikon), by exciting fluorescence using a 488 nm laser (for sfGFP) and a 552 nm laser (for mRuby2/mRuby3). Images were acquired using an Andor iXon EMCCD camera at −80 °C. NIS Elements Advanced Research v.4.5 (Nikon) and Micro-Manager v.2.0 beta were used to control the microscopes.

Macrocolony biofilms of *E. coli* strain (KDE1469) were imaged using the microscope setup described above, but with a ×4/0.2 NA air objective, exciting the constitutively produced sfGFP. Macrocolony biofilms of *E. coli* expressing mRuby2 constitutively (Fig. 2b) were imaged using the spinning-disk confocal microscope described above, equipped with a 552 nm laser. Images were acquired after removing the lid of the Petri dishes, using a ×20/0.4 NA air objective and a *z* spacing of 1 µm, all within a microscope incubator kept at 28 °C.

*B. subtilis* microcolonies (Fig. 1e) were imaged using a ×100/1.4 NA oil-immersion objective on the spinning-disk confocal microscope described above. Free-floating *B. subtilis* aggregates (Fig. 1a) were imaged using a ×40/1.3 NA oil-immersion objective and the spinning-disc confocal microscope, after spotting the culture onto a cover slip. To image a population of three mixed strains of *V. cholerae* (Extended Data Fig. 2f) and to image pellicle biofilms of *B. subtilis* (Fig. 1a) with different fluorescent reporters, images were captured using a Zeiss LSM 880 point-scanning confocal laser scanning microscope with a ×40/1.2 NA water-immersion objective.

*P. aeruginosa* biofilms were imaged using a Leica SP8 confocal microscope with a ×40/1.1 NA water-immersion objective and a *z* step of 3 µm.

### Image analysis

BiofilmQ image analysis is based on a graphical user interface. A stand-alone version of BiofilmQ that does not require a MATLAB license or any interaction with the code is provided. However, as BiofilmQ is open source software written in MATLAB versions R2017b and R2019b (MathWorks), it is possible to adapt BiofilmQ to particular user requirements. Algorithms used for biofilm preprocessing, segmentation, parameter quantification and data visualization are described in detail with examples in the documentation provided online (https://drescherlab.org/data/biofilmQ). All code is freely available, revealing the exact implementation of each data analysis step.

Although BiofilmQ includes in-built options for biofilm semantic segmentation that are useful for many image types, it is also possible to import segmentations prepared by other software tools to enable compatibility with the currently rapidly improving image segmentation results based on machine learning. Thus, the primary focus of BiofilmQ is not to provide the optimal semantic segmentation for microbial community images of all types and all signal levels. Instead, the focus of BiofilmQ is the community property quantification, analysis and data visualization after the segmentation step.

BiofilmQ can analyse not only 3D images, but also 2D images (for example, from epi-fluorescence or confocal microscopy). During the analysis of 2D images, the biofilm segmentation area is dissected into small squares, analogous to the cubes in 3D images, followed by similar analysis and visualization steps to the 3D datasets.

To avoid biases due to optical aberrations and signal blurring along the *z* axis—an artefact that is frequently observed in 3D imaging—a 3D deconvolution before image analysis may be beneficial as a preprocessing step before loading the images into BiofilmQ.

### Data visualization using BiofilmQ

A major functionality of BiofilmQ is the ability to generate various plot types for the data that have been quantified during the image-analysis steps. The different types of data visualization are described below and illustrated in Extended Data Fig. 2. In a spatiotemporal kymograph, the spatial dependence of a biofilm internal property (for example, a fluorescent reporter or any other cube parameter) can be visualized over time (Extended Data Fig. 2b). Importantly, different biofilm internal spatial measures, such as distance-to-surface or distance-to-substrate, can be chosen on the *y* axis for these heat maps, and different temporal measures, such as biofilm volume, can also be used instead of time on the *x* axis. To visualize a global biofilm property as a function of time (Extended Data Fig. 2c) or any other parameter, simple 2D scatterplots with parameter averaging per time frame may be used. There are also options for 2D or 3D scatterplots that do not perform averaging per time frame and can therefore be used with the axes chosen more freely (for example, including spatial coordinates), which also permits a colour-coding of each data point according to another parameter (Extended Data Fig. 2d). Analogous to flow cytometry, the biofilm image cytometry provided by BiofilmQ enables users to apply gates/filters to their data for each cube, to effectively select cube subpopulations with certain characteristics (Extended Data Fig. 2d, inset). The different gated populations can then be analysed separately using the BiofilmQ plotting capabilities. Rendered 3D images, in which each cube parameter can be mapped as colour onto the rendered biofilm biovolume (Extended Data Fig. 2f), can be generated by exporting the BiofilmQ image analysis results into VTK files, which can then be loaded into the open-source 3D-rendering software ParaView^35^.

### Flow cytometry

After imaging the *E. coli* and *B. subtilis* colonies using microscopy in Fig. 1e,f, the colonies were collected by washing them from the agar surface using 1 ml phosphate-buffered saline (PBS). Each resuspended colony was transferred into a 2 ml Eppendorf tube. To disrupt residual cell aggregates, two sterile glass beads (diameter, 4 mm) were added to the tube and the sample was vortexed for 1 min. The sample was then diluted 1:50 in PBS and filtered through a 20 µm filter before flow cytometry analysis using a BD LSRFortessa instrument and the BD FACSDiva software (BD Biosciences). No gating was applied during data collection and analysis. In total 5 × 10^4^ events were acquired in each condition, using 488 nm and 561 nm laser lines for excitation of the green and red fluorescent proteins, respectively.

In these flow cytometry experiments, for which the colonies were resuspended to separate the cells, flow cytometry detected non-fluorescent cell populations of which we observed no evidence in bright-field and fluorescence microscopy. This could be due to false-negative detection by flow cytometry or the existence of cells expressing neither of the fluorescence reporters outside the field of view of the microscopy images analysed.

### Reporting Summary

Further information on research design is available in the Nature Research Reporting Summary linked to this article.

## Supplementary information

Supplementary Information Supplementary Tables 1–6, Notes 1–3 and Discussion.

Reporting Summary

## Data Availability

Test data for exploring BiofilmQ are available at https://drescherlab.org/data/biofilmQ/docs/usage/installation.html. Further image data and processed data used in this study are available from the corresponding author on reasonable request. Source data are provided with this paper.

## Source data

Source Data Fig. 2 Source data for graphs in Fig. 2.

## Code availability

The BiofilmQ documentation, test data, complete source code and a standalone version of BiofilmQ that does not require a MATLAB license are available at https://drescherlab.org/data/biofilmQ. The software download is also mirrored in a GitHub repository (https://github.com/knutdrescher/BiofilmQ). The documentation provides a detailed description of the usage of BiofilmQ, including download and installation, file input, image preparation, image segmentation, parameter calculation, cube tracking, data export, data visualization and plotting, and batch processing and data compatibility with the COMSTAT tool. Furthermore, links to tutorial videos are provided (a list of which is provided in Supplementary Table 6). BiofilmQ and the documentation website will be routinely maintained and updated, initially for five years by H.J., E.J. and N.N. in K.D.’s laboratory. We encourage the use of the BiofilmQ forum (https://forum.image.sc/tags/biofilmq) for reporting bugs and other BiofilmQ-related questions.
