# Publisher Correction: Quantitative image analysis of microbial communities with BiofilmQ

**DOI:** 10.1038/s41564-021-00863-6

**Published:** 2021-01-08

**Authors:** Raimo Hartmann, Hannah Jeckel, Eric Jelli, Praveen K. Singh, Sanika Vaidya, Miriam Bayer, Daniel K. H. Rode, Lucia Vidakovic, Francisco Díaz-Pascual, Jiunn C. N. Fong, Anna Dragoš, Olga Lamprecht, Janne G. Thöming, Niklas Netter, Susanne Häussler, Carey D. Nadell, Victor Sourjik, Ákos T. Kovács, Fitnat H. Yildiz, Knut Drescher

**Affiliations:** 1 Max Planck Institute for Terrestrial Microbiology, Marburg, Germany; 2 Department of Physics, Philipps-Universität Marburg, Marburg, Germany; 3 Department of Microbiology and Environmental Toxicology, University of California, Santa Cruz, CA USA; 4 Bacterial Interactions and Evolution Group, Department of Biotechnology and Biomedicine, Technical University of Denmark, Kongens Lyngby, Denmark; 5 Institute for Molecular Bacteriology, TWINCORE, Centre for Experimental and Clinical Infection Research, Hannover, Germany; 6 Department of Clinical Microbiology, Copenhagen University Hospital, Rigshospitalet, Copenhagen, Denmark; 7 Department of Biological Sciences, Dartmouth College, Hanover, NH USA; 8 Zentrum für Synthetische Mikrobiologie, SYNMIKRO, Marburg, Germany; 9 Present Address: Eawag, Swiss Federal Institute of Aquatic Science and Technology, Dubendorf, Switzerland

**Keywords:** Biofilms, Microbial communities, Computational platforms and environments, Image processing

Correction to: *Nature Microbiology* 10.1038/s41564-020-00817-4, published online 4 January 2021.

In the version of this Brief Communication originally published, in Fig. 1e, the cytometry data were mistakenly omitted from all three graphs. This error has now been corrected and the updated figure is available online.

