# Supplementary material for: Quantitative image analysis of microbial communities with BiofilmQ
Source: Nat Microbiol. 2021 Jan 4;6(2):151–6. doi: 10.1038/s41564-020-00817-4 (PMC7840502; doi:10.1038/s41564-020-00817-4)
Supplement: Supplementary file 1 — Supplementary Tables 1–6, Notes 1–3 and Discussion. [file 41564_2020_817_MOESM1_ESM.pdf]

nature research

https://doi.org/10.1038/s41564-020-00817-4

Supplementary information

Quantitative image analysis of microbial communities with BiofilmQ

In the format provided by the authors and unedited

Supplementary Information

Quantitative image analysis of microbial communities with BiofilmQ

Raimo Hartmann[1,*], Hannah Jeckel[1,2,*], Eric Jelli[1,2,*], Praveen K. Singh[1], Sanika Vaidya[1], Miriam Bayer[1], Daniel K.H. Rode[1,2], Lucia Vidakovic[1], Francisco Díaz-Pascual[1], Jiunn C.N. Fong[3], Anna Dragoš[4], Olga Besharova[1], Janne G. Thöming[5,6], Niklas Netter[1,2], Susanne Häussler[5,6], Carey D. Nadell[7], Victor Sourjik[1,8], Ákos T. Kovács[4], Fitnat H. Yildiz[3], Knut Drescher[1,2,8,#]

[1] Max Planck Institute for Terrestrial Microbiology, Marburg, Germany.

[2] Department of Physics, Philipps-Universität Marburg, Germany.

[3] Department of Microbiology and Environmental Toxicology, University of California, Santa Cruz, CA, USA.

[4] Bacterial Interactions and Evolution Group, Department of Biotechnology and Biomedicine, Technical University of Denmark, Kongens Lyngby, Denmark.

[5] Institute for Molecular Bacteriology, TWINCORE, Centre for Experimental and Clinical Infection Research, Hannover, Germany.

[6] Department of Clinical Microbiology, Copenhagen University Hospital – Rigshospitalet, Copenhagen, Denmark.

[7] Department of Biological Sciences, Dartmouth College, Hanover, NH, USA.

[8] Zentrum für Synthetische Mikrobiologie SYNMIKRO, Marburg, Germany.

* These authors contributed equally.

[#] Correspondence to: k.drescher@mpi-marburg.mpg.de

Supplementary Tables 1-6
Supplementary Discussion
Supplementary Notes 1-3
Supplementary References 1-16

Supplementary Table 1. Biofilm-internal spatially-resolved parameters quantified by BiofilmQ. These parameters are properties calculated for each individual cube object. The table below summarizes all local properties that can be determined using BiofilmQ.

**Parameter name**	**Parameter description**
Architecture_LocalDensity_rangeR	Occupied volume fraction in a sphere of radius R around the object centroid
Architecture_LocalNumberDensity _rangeR	Number of object centroids present in a sphere of radius R around the object
Architecture_LocalSubstrateArea	Contact area of the object with the substrate (in µm²)
BoundingBox	Coordinates and dimensions of the object's bounding box
Centroid	Centre of mass coordinates of the object
Correlation_DensityCorrelation _Binary_chX_chY_rangeR	Only applicable to merged data: Density correlation based on channel X and Y calculated with the images cropped to size R³ such that the merged object is central in the cropped images
Correlation_Local3dOverlap _chY_chX	Biovolume (in µm³) segmented in channel Y that overlaps with the object (in channel X)
Correlation_LocalOverlapFraction _chY_chX	Biovolume segmented in channel Y that overlaps with the object (in channel X) divided by the object´s volume
Correlation_MandersSplit _chX_chY_rangeR	Manders split correlation coefficient [1] of channel Y in channel X calculated with the images cropped to size R³ such that the object is central in the cropped images
Correlation_Manders _chX_chY_rangeR	Manders correlation coefficient based on channel X and Y calculated with the images cropped to size R³ such that the object is central in the cropped images
Correlation_Pearson _chX_chY_rangeR	Pearson correlation coefficient based on channel X and Y calculated with the images cropped to size R³ such that the object is central in the cropped images
Cube_CenterCoord	Center coordinates of the object's cube
Cube_Overlap3D_chY_chX	Calculated during merging of channels: Fraction of biovolume that overlaps between channel X and Y to the merged biovolume given in percentage
Cube_RelativeAbundance_chX	Calculated during merging of channels: Proportion of biovolume in channel X to the combined biovolume given in percentage
Cube_Surface	Surface area of the object (in pixel)
Cube_VolumeFraction	Filling fraction of the object's cube
Distance_ToBiofilmCenterAtSubstrate	Distance of the object to the centre of mass of the biofilm projected onto the substrate (in µm)
Distance_ToBiofilmCenterOfMass	Distance of the object to the centre of mass of the biofilm (in µm)
Distance_ToNearestObject_chX	Centroid-centroid distance of the object to the closest different object in another or the same channel (in µm)
Distance_ToObject_idZ	Centroid-centroid distance of the object to another specific object identified by its ID Z (in µm)
Distance_ToSurface_resolutionR	Distance of the object to the biofilm surface, where the surface shape is smoothed based on the resolution R (in µm)
Grid_ID	Unique ID for each grid point. For two images of the same size and cube side length, the Grid_ID can be identified
ID	Unique ID for each object
Intensity_Integrated_chX	Sum of the intensity values of all pixels of the object
Intensity_Integrated_chX _noBackground	Sum of the intensity values of all pixels of the object after removing a constant value describing the background fluorescence

Intensity_Mean_chX	Mean of the intensity values of all pixels of the object
Intensity_Mean_chX _noBackground	Mean of the intensity values of all pixels of the object after removing a constant value describing the background fluorescence
Intensity_Ratio_Integrated _chY_chX	Sum of the pixelwise ratio of intensity values between two channels X and Y over all pixels of the object
Intensity_Ratio_Integrated _chY_chX_noBackground	Sum of the pixelwise ratio of intensity values between two channels X and Y after removing background fluorescence for each channel over all pixels of the object
Intensity_Ratio_Mean_chY_chX	Mean of the pixelwise ratio of intensity values between two channels X and Y over all pixels of the object
Intensity_Ratio_Mean_chY_chX _noBackground	Mean of the pixelwise ratio of intensity values between two channels X and Y after removing background fluorescence for each channel over all pixels of the object
Intensity_Shells_Integrated_chX _rangeR	Sum of the intensity values in channel X of pixels in a shell of thickness R around the object
Intensity_Shells_Integrated_ noBackground_chX_rangeR	Sum of the intensity values in channel X of pixels in a shell of thickness R around the object after removing background
Intensity_Shells_Mean_chX_rangeR	Mean of the intensity values in channel X of pixels in a shell of thickness R around the object
Intensity_Shells_Mean_ noBackground_chX_rangeR	Mean of the intensity values in channel X of pixels in a shell of thickness R around the object after removing background
Shape_Convexity	Volume of the object divided by the volume of the convex hull of the object
Shape_Volume	Volume of the object (in μm^3)
Surface_LocalThickness	Thickness of the biofilm at the x-y-position of the object (in µm)
Surface_PerSubstrateArea	Combined surface area of objects sharing the same Cube_CenterCoord normalized by their x-y-planar area
Texture_Haralick_ClusterShade_ chX_rangeR	Haralick Cluster Shade, calculated based on Haralick *et al.* [2], with the image cropped to size R^3 such that the object is central in the cropped images.
Texture_Haralick_ClusterTendendy_ chX_rangeR	Haralick Cluster Tendency
Texture_Haralick_Contrast_ chX_rangeR	Haralick Contrast
Texture_Haralick_Correlation_ chX_rangeR	Haralick Correlation
Texture_Haralick_Energy_chX_ rangeR	Haralick Energy
Texture_Haralick_Entropy_chX_ rangeR	Haralick Entropy
Texture_Haralick_Homogeneity_chX_ rangeR	Haralick Homogeneity
Texture_Haralick_Inertia_chX_ rangeR	Haralick Inertia
Texture_Haralick_InverseVariance_ chX_rangeR	Haralick Inverse Variance
Texture_Haralick_MaxProbability_ chX_rangeR	Haralick Maximal Probability
Texture_Haralick_SumMean_chX_ rangeR	Haralick Sum Average
Texture_Haralick_Variance_chX_ rangeR	Haralick Variance

Supplementary Table 2. Global biofilm parameters quantified by BiofilmQ. Global parameters are calculated for the entire biofilm, based on all segmented objects in the image.

Parameter name	Parameter description
Biofilm_AspectRatio_HeightToLength	Ratio between Biofilm_Height and Biofilm_Length
Biofilm_AspectRatio_HeightToWidth	Ratio between Biofilm_Height and Biofilm_Width
Biofilm_AspectRatio_LengthToWidth	Ratio between Biofilm_Length and Biofilm_Width
Biofilm_AutoCorrelation_ CorrelationFcn_chX	Matlab structure containing the 2D and 3D autocorrelation function
Biofilm_AutoCorrelation_ CorrelationLength2D_Substrate_chX	2D autocorrelation length at the substrate layer (brightest image plane)
Biofilm_AutoCorrelation_ CorrelationLength2D_chX	2D autocorrelation length based on the mean over all layers
Biofilm_AutoCorrelation_ CorrelationLength3D_chX	3D autocorrelation length
Biofilm_AutoCorrelation_Zero2D _Substrate_chX	Position of first zero-crossing of the 2D autocorrelation function at the substrate layer
Biofilm_AutoCorrelation_Zero2D_chX	Position of first zero -crossing of the 2D autocorrelation function mean
Biofilm_AutoCorrelation_Zero3D_chX	Position of first zero -crossing of the 3D autocorrelation function
Biofilm_BaseArea	Area of an ellipsoid fitted to the convex hull of the biofilm projected onto the x-y-plane
Biofilm_BaseEccentricity	Eccentricity of an ellipsoid fitted to the convex hull of the biofilm projected onto the x-y-plane
Biofilm_Correlation_MandersSplit _chY_chX	Manders split correlation coefficient based on the complete image
Biofilm_Correlation_Manders_chX_chY	Manders correlation coefficient for channels X and Y based on the complete image
Biofilm_Correlation_Pearson_chX_chY	Pearson correlation coefficient for channels X and Y based on the complete image
Biofilm_Height	The difference between the 99.5 percentile and 1 percentile of the z-coordinates of cells
Biofilm_Length	Major axis length of an ellipsoid fitted to the convex hull of the biofilm projected onto the x-y-plane
Biofilm_MeanThickness	Mean of the local parameter Surface_LocalThickness
Biofilm_OuterSurface	Surface Area of the biofilm (in pixel)
Biofilm_OuterSurface_ignoreSubstrate	Surface area of the biofilm, excluding pixels in the lowest plane of the biofilm
Biofilm_OuterSurfacePerSubstrate	Biofilm_OuterSurface divided by Biofilm_SubstrateArea
Biofilm_OuterSurfaceIgnoreSubstratePerSubstrate	Biofilm_OuterSurface_ignoreSubstrate divided by Biofilm_SubstrateArea
Biofilm_OuterSurfacePerVolume	Biofilm_OuterSurface divided by Biofilm_ Volume
Biofilm_OuterSurfaceIgnoreSubstratePerVolume	Biofilm_OuterSurface_ignoreSubstrate divided by Biofilm_Volume
Biofilm_OverlapFraction_chX_chY	Mean of the local parameter Correlation_LocalOverlapFraction_chY_chX
Biofilm_Overlap_chX_chY	Sum of the local parameter Correlation_Local3dOverlap_chY_chX
Biofilm_Roughness	Biofilm roughness defined as the mean of the difference between local thickness and mean thickness divided by mean thickness

Biofilm_SubstrateArea	Sum of the local parameter Architecture_LocalSubstrateArea (in μm^2) if present, otherwise equal to Biofilm_BaseArea
Biofilm_SubstratumCoverage	Between 0 and 1, quantifies the fraction of substratum that is covered by microbial cells
Biofilm_Volume	Combined volume of all segmented objects (in μm^3)
Biofilm_VolumePerSubstrate	Biofilm_Volume divided by Biofilm_SubstrateArea
Biofilm_Width	Minor axis length of an ellipsoid fitted to the convex hull of the biofilm projected onto the x-y-plane

Supplementary Table 3. Additional global biofilm parameters quantified from each local parameter. Based on each local parameter (which are parameters quantified for each cube, see Supplementary Table 1), BiofilmQ can compute 27 global parameters from the values of all cubes in the biofilm, or from the values of only those cubes in particular regions inside the biofilm. With “particular regions” in the biofilm, we are referring to the biofilm core (corresponding to spatial locations deep below the biofilm outer surface), and the biofilm shell (corresponding to spatial locations close to the biofilm outer surface). Additionally, the average and standard deviation of each cube parameter is calculated (non-weighted and weighted by the cube’s biovolume in relation to the full biofilm biovolume), and the 25 and 75 percentiles are calculated in the parameters with the suffix “_p25” and “_p75”, respectively. The 27 parameters that are derived from a fictitiously named local parameter “InternalParameterName” are listed below. An analogous set of derived parameters is computed for each of the local parameters listed in Supplementary Table 1.

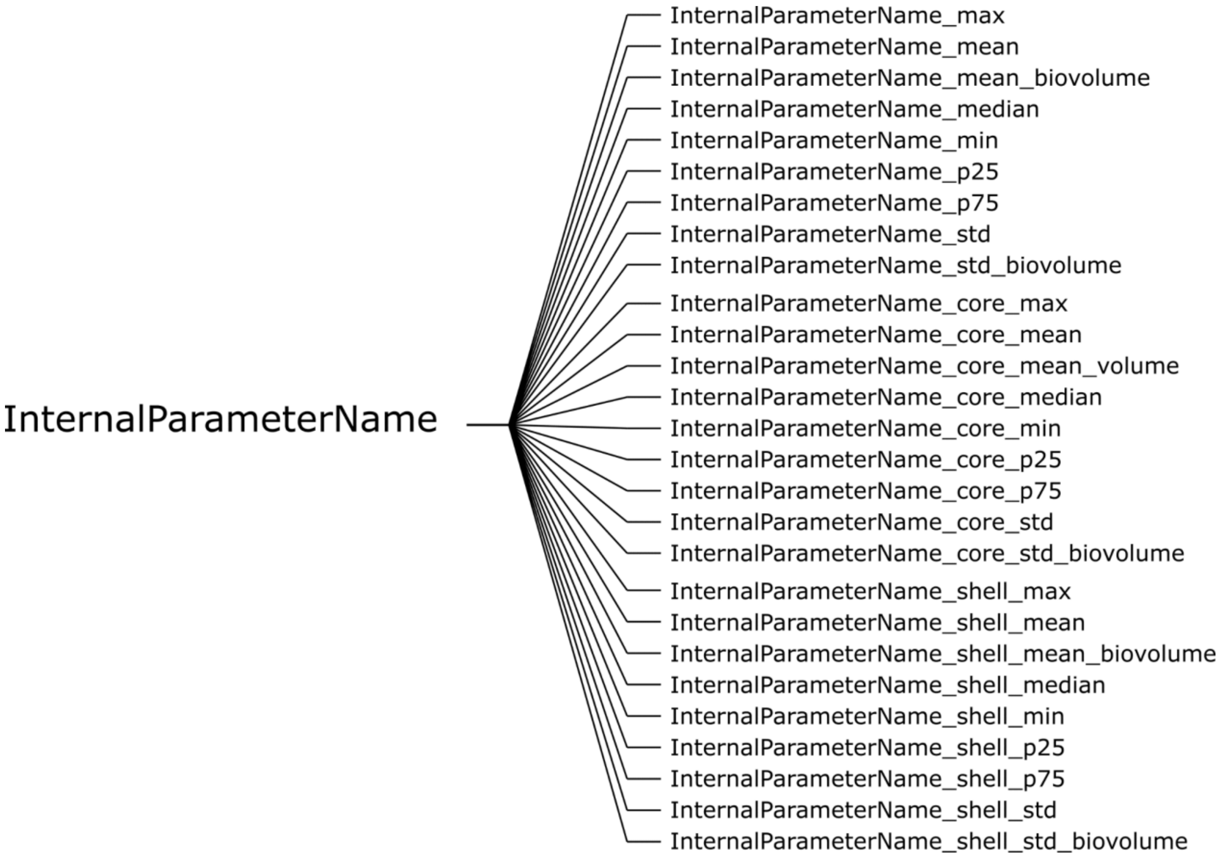

Supplementary Table 4. Compatibility of BiofilmQ with COMSTAT parameters.

COMSTAT parameter	BiofilmQ parameter (exact match)	Related parameters in BiofilmQ	Comments
Global and local parameters			
Bio-volume		Biofilm_Volume	Parameters differ by a constant factor (image size)
Substratum coverage	Biofilm_SubstratumCoverage		Index of substrate can be freely chosen in BiofilmQ. In COMSTAT it is set to 1.
Thickness distribution	Surface_LocalThickness		Identical measures can be achieved with BiofilmQ if cube side length is set to 1 pixel.
Mean biofilm thickness	Biofilm_meanThickness		Identical measures can be achieved with BiofilmQ if cube side length is set to 1 pixel.
Maximal biofilm thickness	Surface_LocalThickness_max		Identical measures can be achieved with BiofilmQ if cube side length is set to 1 pixel.
Surface area	Biofilm_OuterSurface_ignoreSubstrate		
Average diffusion distance		DistanceToSurface_ resolutionx_mean	Diffusion distance calculation differs from COMSTAT: In BiofilmQ, a convex hull of the biofilm is calculated prior to distance calculations.
Maximal diffusion distance		DistanceToSurface_ resolutionx_max	
Biofilm roughness	Biofilm_Roughness		
Surface to volume ratio		Biofilm_ OuterSurfaceToVolume	Parameters differ by a constant factor (image size)
Micro-colony parameters/properties			
Number of micro-colonies	Cell_Number		
Volume of micro-colonies	Shape_Volume		
Mean volume of micro-colonies	Shape_Volume_mean		
Number of micro-colonies at substrate			Can be achieved in BiofilmQ by counting the micro-colonies with substrate area greater than 0.
Substrate area of micro-colonies	Architecture_LocalSubstrateArea		
Mean substrate area of micro-colonies	Architecture_ LocalSubstrateArea_mean		

Supplementary Table 5. Bacterial and viral strains used in this study.

Strain name	Genotype/characteristics	Origin/reference
Bacteria		
KDV201	*V. cholerae* C6706 (O1 El Tor, Sm^R) wild type	[1]
KDV101	*V. cholerae* C6706 (O1 El Tor, Sm^R) wild type	[2]
KDV323	*V. cholerae* N16961 WT contains plasmid pNUT546	Drescher lab stock
KDV371	*V. cholerae* N16961 *lacZ*:P_{tac}-mRuby2, contains plasmid pNUT546	Drescher lab stock
KDV613	*V. cholerae* N16961 Rugose, Δ*crvA* contains plasmid pNUT542	[3]
KDV903	*V. cholerae* C6706 *rpoS*::4aa-mRuby3 contains plasmid pNUT542	[4]
KDV1027	*V. cholerae* N16961 *vpvC*^{W240R}, Δ*crvA, rbmA:mRuby3* and plasmid pNUT542	[3]
KDV2229	*V. cholerae* C6706 *rbmA-mRuby3* transcriptional fusion, and plasmid pNUT542	This study
KDV2756	*V. cholerae* C6706 carrying *rbmC*-reporter plasmid pNUT2453	This study
KDV2755	*V. cholerae* C6706 carrying *bap1*-reporter plasmid pNUT2452	This study
KDV2151	*V. cholerae* C6706 *rbmA*-6xHis Tag	This study
KDV2192	*V. cholerae* C6706 *rbmC*-6xHis Tag	This study
KDV2186	*V. cholerae* C6706 *bap1*-6xHis Tag	This study
KDV115	*V. cholerae* N16961, *vpvC*^{W240R}, *lacZ*:P_{tac}-*mKOkappa*:*lacZ*	[5]
KDV116	*V. cholerae* N16961, *vpvC*^{W240R}, *lacZ*:P_{tac}-*mKate2*:*lacZ*	[5]
	V. cholerae N16961, *vpvC*^{W240R}, *lacZ*:P_{tac}-*mKOkappa*:*lacZ*	Carey Nadell lab stock
KDE474	*E. coli AR3110 WT*	[6]
KDE679	*E. coli* AR3110, with P*tac _mRuby2* and Kan^R inserted at *attB* site (P*tac* without operator)	[7]
KDE1469	*E. coli* AR3110, P_{tac} -sfGFP and Kan^R inserted at the *attB* site	This study
KDE542	*E. coli* MG1655, with pNUT542	This study
KDE1029	*E. coli* MG1655, with pNUT1029	This study
TB960	*B. subtilis* DK1042 (NCIB3610 *comI*^{Q12I}) *amyE*::$P_{tapA\_}$*mKate* (Cm^R), *sacI*::P_{eps}*_gfp* (Km^R)	[8]
KDB026	*B. subtilis* NCIB3610 Δ*comI, amyE::Physpank-sfGFP*	This study
KDB017	*B. subtilis* 168*, amyE::Physpank-sfGFP*	This study
KDB174	*B. subtilis* 168*, lacA::Pspank-mKate2*	This study
694 clinical isolates of *P. aeruginosa*	The different *P. aeruginosa* clinical isolates are characterized in the BACTOME database.	[9,10] and this study
Plasmids		
pNUT546	p15AOri, $Gent^R$, P_{tac}-*sfGFP*	Drescher lab stock
pNUT542	pSC101* Ori, $Gent^R$, P_{tac}-*sfGFP*	[4]
pNUT1029	pSC101* Ori, $Gent^R$, P_{tac}-*mRuby3*	This study
pNUT2452	p15A Ori, $Gent^R$, P_{bap1}-*mRuby3, P_{tac}-gfp*	This study
pNUT2453	p15A Ori, $Gent^R$, P_{rbmC}-*mRuby3, P_{tac}-gfp*	This study

Supplementary Table 6. List of video tutorials for BiofilmQ.

Title	URL
1. Introduction to BiofilmQ	https://www.youtube.com/watch?v=h9LQFJuDzC0&t=8s
1.1 Installation	https://www.youtube.com/watch?v=pmNLJQI3ryM&t=6s
1.2 General Overview of BiofilmQ	https://www.youtube.com/watch?v=oIHdAKxe4v8&t=6s
1.3.1 Import microscope image files	https://www.youtube.com/watch?v=amNzczgsHDI&t=5s
1.3.2 Import custom TIF files	https://www.youtube.com/watch?v=0yECmC9tTkM&t=4s
2. Image Processing steps	https://www.youtube.com/watch?v=pNTgBIUj_iA&t=8s
2.1 Image Preparation	https://www.youtube.com/watch?v=m9KgHJNZs2E&t=6s
2.1.1 Colony Separation	https://www.youtube.com/watch?v=YkfQwYv20Uw&t=7s
2.1.2 Image Alignment	https://www.youtube.com/watch?v=g3zSGuWvrfE&t=1s
2.2 Segmentation Overview	https://www.youtube.com/watch?v=JKi3n4Fqdf8&t=7s
2.2.1 Image Cropping	https://www.youtube.com/watch?v=8vLSU_hAwL4&t=5s
2.2.2 Denoising	https://www.youtube.com/watch?v=EX5gOnhi9Co&t=7s
2.2.3 Thresholding	https://www.youtube.com/watch?v=kGwFJkXe0Lw&t=5s
2.3 Parameter Calculation	https://www.youtube.com/watch?v=GqVr_G95VSY&t=6s
2.3.1 Fluorescence properties	https://www.youtube.com/watch?v=HyMbjmJmx7I&t=7s
2.4 Cube Tracking	https://www.youtube.com/watch?v=xB2wNUxMJUg&t=7s
2.5 Data Export	https://www.youtube.com/watch?v=9etbgANoj1k&t=4s
3. Visualization Overview	https://www.youtube.com/watch?v=bC__dewjTyg&t=14s
3.1.1 Plotting Options	https://www.youtube.com/watch?v=1z_Ekdi7q4c&t=2s
3.1.2 Histograms	https://www.youtube.com/watch?v=acDUKapP-_I
3.1.3 2D XY-plot per time point/frame	https://www.youtube.com/watch?v=tjjDwyS3Hno&t=1s
3.1.4 Kymograph & Demograph	https://www.youtube.com/watch?v=yoSqEyAWhIg&t=3s
3.1.5 Scatter Plots	https://www.youtube.com/watch?v=G9Mt2NC3mro&t=2s
3.2 Visualization Filtering	https://www.youtube.com/watch?v=o4e7QzHAo7A&t=2s
3.3 Advanced Visualization options	https://www.youtube.com/watch?v=mVhYyCyqfKM&t=2s
4.1 Micro-Colony Analysis	https://www.youtube.com/watch?v=vqmA8kD1tTQ

Supplementary Discussion

Even for low-resolution images, BiofilmQ enables spatially-resolved cytometry based on the approach of dissecting a biofilm biovolume into small cubes, as long as either (*i*) a biofilm biovolume can be detected in one fluorescent channel using the BiofilmQ-inbuilt segmentation options, or (*ii*) the biofilm biovolume can be detected with another software tool or by manual annotation, followed by an import of the segmented binary images into BiofilmQ. Once the biofilm biovolume is identified, BiofilmQ can quantify many spatially-resolved biofilm properties, and their variation in time series, and the software integrates the visualization of these properties in kymographs, demographs, and other simple and complex plots.

Cube-based image cytometry can also provide important quantitative information for the analysis of the biofilm stress response, as demonstrated by our use of an early cube-cytometry approach for measuring the spatiotemporal dynamics of an RpoS-mRuby3 translational fusion following glucose removal in *Vibrio cholerae* biofilms during dispersal[4]. Similarly, we previously used our early cube-based image cytometry to analyse the interaction dynamics of *E. coli* biofilms with T7 phages[7].

Supplementary Note 1: Key steps in the BiofilmQ analysis workflow

Please note that further documentation and technical details beyond those described below, are provided in the BiofilmQ online documentation (https://drescherlab.org/data/biofilmQ/).

Importing images:
BiofilmQ supports most major microscopy image formats containing multiple imaging positions, z-stacks, fluorescence channels, and time series. But BiofilmQ can also import 2D image sequences to reconstruct 3D z-stacks and time series. BiofilmQ automatically converts the supplied microscopy images into its own internal multipage-TIF-based image data format. Each 3D image time series, or each set of 3D images for investigating biofilm population variation, is treated as a separate project, and BiofilmQ will generate the necessary folder structure for the next analysis steps and data export.

Biofilm semantic segmentation:
The semantic segmentation of the image results in a binary image that defines the biofilm volume for the downstream analysis steps. BiofilmQ includes several options for semantic segmentation algorithms, but users may also import their own pre-segmented binary images into BiofilmQ, *e.g.* by using a general-purpose segmentation tool such as ilastik[11], or a neural network (such as a U-Net[12]) that is trained with the particular dataset of interest to the user. A segmentation can be imported by introducing its binary output image or label image as a new channel in BiofilmQ, on which BiofilmQ's segmentation should then be performed *via* trivial thresholding.

Cube dissection:
Following the detection of the biofilm volume (or the import of a semantic segmentation as described above), the biofilm volume can be dissected into cubes with a user-defined size, as described in the main text. Alternatively, an instance segmentation result from other software tools may be imported, *e.g.* using Mask R-CNN[13] or StarDist[14], in which case the dissection method "None" should be chosen in BiofilmQ.

Quantification of biofilm global parameters, and biofilm-internal local parameters:
"Global parameters" (Fig. 1d, Extended Data Fig. 1d) are those that yield one scalar value for the whole biofilm biovolume. "Local parameters" (Fig. 1c, Extended Data Fig. 1d) are quantified for each cube in the biofilm. A summary of these parameters is given in the main text and above in Supplementary Tables 1-3.

Time series analysis of biofilm images:

The biofilm time series analysis workflow is summarized in Extended Data Fig. 1. Before segmentation, a biofilm time series can be corrected for drifts in position (Extended Data Fig. 1b) by image registration. Once the biofilm has been segmented, global as well as local parameters can be calculated using the parameter calculation tab in the graphical user interface (Extended Data Fig. 1d). In this tab, different parameters are represented by several modules, such as surface properties or fluorescence properties, which can be added or removed from the parameter calculation performed by BiofilmQ, to minimize computation time.

Analysing a population of biofilms:
To analyse and compare a population of biofilms with respect to the global or spatially-resolved local properties, the workflow is identical to the time series analysis, except that image registration is not required. Each analysed biofilm in the population is then assigned an index for the analysis, analogous to a frame index during time series analysis. If multiple biofilms are in the same image, BiofilmQ can analyse them separately (Extended Data Fig. 1b).

Data export:
BiofilmQ routinely saves all data from the image analysis to its own internal Matlab-based file format for the BiofilmQ visualization pipeline. However, it is also possible to export the analysis results, or a user-defined subset of the results, to standard formats (Extended Data Fig. 1e), including the flow cytometry standard FCS-format, and the VTK-format for creating 3D renderings in ParaView, or as CSV-files for spreadsheet software such as Microsoft Excel.

Data visualization:
The key data visualization features of BiofilmQ are summarized in the main text and in the BiofilmQ online documentation.

Supplementary Note 2: Lineage tracking for cubes

BiofilmQ offers the option to track cubes within a time series. Cube tracking is a powerful tool to investigate clonal cluster sizes in experiments with multiple strains which share the same fluorescent label. By acquiring time series starting with well-separated clonal clusters, the strains can still be separated using the cube tracking algorithm even if the clones share the same fluorescent label.

For each cube lineage, a separate *TrackID* variable is assigned. For two consecutive time frames, the ancestor cube of a cube in the current time frame is called *Parent*. The ancestor of the parent is called *Grandparent*. A growth rate is calculated by comparing the volume of all objects originating from the same parent with the volume of the parent itself and dividing by the number of offspring. Additionally, the loss of biovolume (which is relevant if there is biofilm dispersal) is calculated and assigned to the nearest remaining cube.

The tracking algorithm has 3-4 different stages. At each stage, a different method is used to find a (grand-)parent cube.

1. Test whether a cube position was occupied at the previous time point. If yes, assign previous cube as *Parent*.
2. Test whether a cube position was occupied two time points before the current time point. If yes, assign the object as *Grandparent* and set the *Parent* parameter to the Matlab value NaN.
3. If steps 1 and 2 do not yield a parent/grandparent, search the distance defined by the search radius (which users can specify) for cubes in the previous time point.
    a. If only a single cube can be found in the search radius, assign it as new *Parent*.
    b. If multiple objects can be found, calculate the volume overlap of cubes. Users can increase the overlap by dilating each cube by a user-specified dilation size-parameter. Cubes, which are not dilated, usually do not yield any overlap.
        i. If more than one overlapping cubes are found, assign the object with the largest overlap as *Parent*.

    ii. If no overlapping cubes are found, assign the object with the smallest distance within the search radius as *Parent*.

4. (Optional) If the user chooses to assign the same *TrackID* to connected clusters (*e.g.* to track clonal clusters starting from a cluster that is larger than one cube), all cubes that are not yet assigned a *TrackID* are grouped together in the distance defined by the search radius specified under stage 3.
    a. If one of the grouped cubes has a parent, it is assigned as parent of all other cubes in the cluster.
    b. If none of the grouped cubes has a parent, they are all assigned an identical new *TrackID* value.

Supplementary Note 3: Compatibility with COMSTAT

COMSTAT is a biofilm image analysis software tool that has been widely-used, published by Heydorn *et al.*[15] in the year 2000, and updated as COMSTAT2 in 2008[16]. Most COMSTAT parameters can also be calculated using BiofilmQ, to enable compatibility. This Supplementary Note describes how to achieve this. More information is provided in the online documentation of BiofilmQ.

Segmentation: BiofilmQ offers to crop or denoise images by filters prior to segmentation, however, all of these operations are optional. If users would like to perform thresholding on the raw image data, analogous to COMSTAT, BiofilmQ users need to uncheck all of the filtering and cropping options in the segmentation tab of BiofilmQ. The automated COMSTAT thresholding via Otsu's method is also implemented in BiofilmQ. To achieve the same results, BiofilmQ users should navigate to the segmentation tab, select the thresholding tab, choose "Otsu" in the drop-down menu, and choose the option "2" for the number of Otsu segmentation classes, and make sure that the text field "sensitivity" is set to 1. This will result in identical automated thresholding for BiofilmQ and COMSTAT. If users prefer the manual thresholding in COMSTAT, the same functionality is offered by BiofilmQ by navigating to the thresholding tab, and selecting the manual mode in the drop-down menu.

Global and local biofilm parameters: Global parameters are parameters that yield one scalar value for each image file, for example biovolume. Local properties are defined per pixel in COMSTAT and per cube in BiofilmQ. By choosing the cube side length equal to 1 pixel in BiofilmQ, each cube corresponds to a pixel and the parameters will be identical in both the COMSTAT and BiofilmQ implementation. Users should note, however, that a resolution of 1 pixel is often not necessary and the computation time can be decreased dramatically by increasing the cube size. To set the cube size in BiofilmQ, navigate to the segmentation tab, select the declumping method tab, and choose cubes in the drop-down menu, and enter the cube side length in pixel units. Supplementary Table 4 gives an overview of the COMSTAT parameters that have a corresponding implementation in BiofilmQ. To compute these parameters with BiofilmQ, users need to check the quantification modules "substrate area", "surface properties", and "distance to surface" in the parameter calculation tab prior to parameter calculation.

Micro-colony parameters: COMSTAT allows users to calculate properties for each micro-colony in the field of view. To achieve the same results in BiofilmQ, navigate to the segmentation tab and choose the declumping method "none" in the drop-down menu of the object declumping tab. Now, BiofilmQ treats every micro-colony in the field of view like a single object and local parameters no longer refer to a cube, but to a micro-colony instead. With these settings many COMSTAT micro-colony parameters have a counterpart in BiofilmQ, as listed in Supplementary Table 4.

References

1. Thelin, K. H. & Taylor, R. K. Toxin-coregulated pilus, but not mannose-sensitive

hemagglutinin, is required for colonization by Vibrio cholerae O1 El Tor biotype and O139 strains. *Infect. Immun.* **64**, 2853–6 (1996).
2. Meibom, K. L. *et al.* The Vibrio cholerae chitin utilization program. *Proc. Natl. Acad. Sci.* **101**, 2524–2529 (2004).
3. Hartmann, R. *et al.* Emergence of three-dimensional order and structure in growing biofilms. *Nat. Phys.* **15**, 251–256 (2019).
4. Singh, P. K. *et al.* Vibrio cholerae Combines Individual and Collective Sensing to Trigger Biofilm Dispersal. *Curr. Biol.* **27**, 3359-3366.e7 (2017).
5. Drescher, K., Nadell, C. D., Stone, H. A., Wingreen, N. S. & Bassler, B. L. Solutions to the Public Goods Dilemma in Bacterial Biofilms. *Curr. Biol.* **24**, 50–55 (2014).
6. Serra, D. O., Richter, A. M., Klauck, G., Mika, F. & Hengge, R. Microanatomy at cellular resolution and spatial order of physiological differentiation in a bacterial biofilm. *MBio* **4**, e00103-13 (2013).
7. Vidakovic, L., Singh, P. K., Hartmann, R., Nadell, C. D. & Drescher, K. Dynamic biofilm architecture confers individual and collective mechanisms of viral protection. *Nat. Microbiol.* **3**, 26–31 (2018).
8. Dragoš, A. *et al.* Division of Labor during Biofilm Matrix Production. *Curr. Biol.* **28**, 1903-1913.e5 (2018).
9. Thöming, J. G. *et al.* Parallel evolutionary paths to produce more than one Pseudomonas aeruginosa biofilm phenotype. *npj Biofilms Microbiomes* **6**, 1–13 (2020).
10. Hornischer, K. *et al.* BACTOME-a reference database to explore the sequence-and gene expression-variation landscape of Pseudomonas aeruginosa clinical isolates. *Nucleic Acids Res.* **47**, (2019).
11. Berg, S. *et al.* ilastik: interactive machine learning for (bio)image analysis. *Nat. Methods* **16**, 1226–1232 (2019).
12. Ronneberger, O., Fischer, P. & Brox, T. U-net: Convolutional networks for biomedical image segmentation. in *Lecture Notes in Computer Science (including subseries Lecture Notes in Artificial Intelligence and Lecture Notes in Bioinformatics)* **9351**, 234–241 (Springer Verlag, 2015).
13. He, K., Gkioxari, G., Dollár, P. & Girshick, R. Mask R-CNN. *Proc. IEEE Int. Conf. Comput. Vis.* 2961–2969 (2017).
14. Weigert, M., Schmidt, U., Haase, R., Sugawara, K. & Myers, G. Star-convex Polyhedra for 3D Object Detection and Segmentation in Microscopy. *Proc. IEEE/CVF Winter Conf. Appl. Comput. Vis.* 3666–3673 (2020).
15. Heydorn, A. *et al.* Quantification of biofilm structures by the novel computer program COMSTAT. *Microbiology* **146 (Pt 10)**, 2395–407 (2000).
16. Vorregaard, M. Comstat2-a modern 3D image analysis environment for biofilms. (Technical University of Denmark, 2008).
