# Supplementary material for: Quantitative image analysis of microbial communities with BiofilmQ
Source: Nat Microbiol. 2021 Jan 4;6(2):151–6. doi: 10.1038/s41564-020-00817-4 (PMC7840502; doi:10.1038/s41564-020-00817-4)
Supplement: Supplementary file 2 — Reporting Summary [file 41564_2020_817_MOESM2_ESM.pdf]

natureresearch

Corresponding author(s): Knut Drescher

Last updated by author(s): Oct 8, 2020

# Reporting Summary

Nature Research wishes to improve the reproducibility of the work that we publish. This form provides structure for consistency and transparency in reporting. For further information on Nature Research policies, see Authors & Referees and the Editorial Policy Checklist.

## Statistics

For all statistical analyses, confirm that the following items are present in the figure legend, table legend, main text, or Methods section.

| n/a | Confirmed | |
|---|---|---|
| ☐ | ☒ | The exact sample size ($n$) for each experimental group/condition, given as a discrete number and unit of measurement |
| ☐ | ☒ | A statement on whether measurements were taken from distinct samples or whether the same sample was measured repeatedly |
| ☒ | ☐ | The statistical test(s) used AND whether they are one- or two-sided<br>*Only common tests should be described solely by name; describe more complex techniques in the Methods section.* |
| ☒ | ☐ | A description of all covariates tested |
| ☒ | ☐ | A description of any assumptions or corrections, such as tests of normality and adjustment for multiple comparisons |
| ☒ | ☐ | A full description of the statistical parameters including central tendency (e.g. means) or other basic estimates (e.g. regression coefficient) AND variation (e.g. standard deviation) or associated estimates of uncertainty (e.g. confidence intervals) |
| ☒ | ☐ | For null hypothesis testing, the test statistic (e.g. $F$, $t$, $r$) with confidence intervals, effect sizes, degrees of freedom and $P$ value noted<br>*Give P values as exact values whenever suitable.* |
| ☒ | ☐ | For Bayesian analysis, information on the choice of priors and Markov chain Monte Carlo settings |
| ☒ | ☐ | For hierarchical and complex designs, identification of the appropriate level for tests and full reporting of outcomes |
| ☐ | ☒ | Estimates of effect sizes (e.g. Cohen's $d$, Pearson's $r$), indicating how they were calculated |

*Our web collection on statistics for biologists contains articles on many of the points above.*

## Software and code

Policy information about availability of computer code

| | |
|---|---|
| Data collection | Nikon NIS Elements Advanced Research 4.5 software and Micro-Manager 2.0beta were used to control microscopes for image acquisition. |
| Data analysis | This paper presents a new data analysis tool, BiofilmQ. All data analysis was performed with BiofilmQ to demonstrate the technical capabilities of this tool. The source code, standalone executable, and documentation of this tool are available at https://drescherlab.org/data/biofilmQ . In addition, the source code is mirrored to a GitHub repository at https://github.com/knutdrescher/BiofilmQ . The code was developed in Matlab (MathWorks), using Matlab versions R2017b and R2019b. |

For manuscripts utilizing custom algorithms or software that are central to the research but not yet described in published literature, software must be made available to editors/reviewers. We strongly encourage code deposition in a community repository (e.g. GitHub). See the Nature Research guidelines for submitting code & software for further information.

## Data

Policy information about availability of data

All manuscripts must include a data availability statement. This statement should provide the following information, where applicable:

- Accession codes, unique identifiers, or web links for publicly available datasets
- A list of figures that have associated raw data
- A description of any restrictions on data availability

Source data used for figures are available in the Supplementary Information. Test data for exploring BiofilmQ are available at https://drescherlab.org/data/biofilmQ/docs/usage/installation.html . Further image data and processed data used in this study are available from the corresponding author upon reasonable request.

# Field-specific reporting

Please select the one below that is the best fit for your research. If you are not sure, read the appropriate sections before making your selection.

☒ Life sciences ☐ Behavioural & social sciences ☐ Ecological, evolutionary & environmental sciences

For a reference copy of the document with all sections, see nature.com/documents/nr-reporting-summary-flat.pdf

# Life sciences study design

All studies must disclose on these points even when the disclosure is negative.

| | |
|---|---|
| Sample size | In figures that show example data analyzed with BiofilmQ, the sample size (n, corresponding to the number of different biofilm colonies) for each experiment is indicated in the caption. Sample sizes were chosen to illustrate the functionality and capabilities of the BiofilmQ software, using 3 independent replicate experiments, which was determined to be sufficient if all replicates showed the same trend. For the screening of 694 wild isolates of Pseudomonas aeruginosa (Fig. 2c) it was technically not possible to perform the screen three independent times, so that fewer replicates are available, as indicated in the caption. |
| Data exclusions | No data were excluded. |
| Replication | The manuscript presents a data analysis and visualization method. The number (n) of biologically independent replica biofilms that are analyzed in each graph are provided. Each experiment was performed three times independently successfully, resulting in the same qualitative result. Only the Pseudomonas aeruginosa screen was performed only once, but with multiple biofilms imaged for each strain. |
| Randomization | This manuscript shows only example data, without drawing biological conclusions from these data so that sample randomization is irrelevant for the manuscript data. |
| Blinding | Blinding of group allocation is irrelevant to our data analysis, because there was no allocation to experimental groups, beyond collecting n replicates, all of which were analyzed by software equally. |

# Reporting for specific materials, systems and methods

We require information from authors about some types of materials, experimental systems and methods used in many studies. Here, indicate whether each material, system or method listed is relevant to your study. If you are not sure if a list item applies to your research, read the appropriate section before selecting a response.

## Materials & experimental systems

| n/a | Involved in the study | |
|---|---|---|
| ☒ | ☐ | Antibodies |
| ☒ | ☐ | Eukaryotic cell lines |
| ☒ | ☐ | Palaeontology |
| ☒ | ☐ | Animals and other organisms |
| ☒ | ☐ | Human research participants |
| ☒ | ☐ | Clinical data |

## Methods

| n/a | Involved in the study | |
|---|---|---|
| ☒ | ☐ | ChIP-seq |
| ☐ | ☒ | Flow cytometry |
| ☒ | ☐ | MRI-based neuroimaging |

## Flow Cytometry

### Plots

Confirm that:

☒ The axis labels state the marker and fluorochrome used (e.g. CD4-FITC).

☒ The axis scales are clearly visible. Include numbers along axes only for bottom left plot of group (a 'group' is an analysis of identical markers).

☒ All plots are contour plots with outliers or pseudocolor plots.

☒ A numerical value for number of cells or percentage (with statistics) is provided.

### Methodology

| | |
|---|---|
| Sample preparation | After imaging the E. coli and B. subtilis colonies by microscopy in Fig. 1e, f, the colonies were collected by washing them from the agar surface using 1 ml phosphate-buffered saline (PBS). Each resuspended colony was transferred into a 2 ml Eppendorf tube. To disrupt residual cell aggregates, two sterile glass beads (4 mm diameter) were added to the tube and the sample was vortexed for 1 minute. The sample was then diluted 1:50 in PBS and filtered through a 20 μm filter prior to the flow cytometry analysis. |

| | |
|---|---|
| Instrument | BD LSRFortessa instrument (BD Biosciences). |
| Software | BD FACSDiva software was used to collect data. Plots were generated in Matlab R2019b without postprocessing. |
| Cell population abundance | The whole colony population was analyzed for each flow cytometry experiment, no sorting and no gating was applied. The number of cells is indicated in the plots. |
| Gating strategy | No gating was applied during data collection and analysis, as mentioned in the methods section of the manuscript. |

☐ Tick this box to confirm that a figure exemplifying the gating strategy is provided in the Supplementary Information.
